# Laparoscopic Reversal of Hartmann's Procedure: State of the Art 20 Years after the First Reported Case

**DOI:** 10.1155/2014/530140

**Published:** 2014-08-21

**Authors:** Adriana Toro, Annalisa Ardiri, Maurizio Mannino, Antonio Politi, Andrea Di Stefano, Zia Aftab, Abdelrahman Abdelaal, Maria Concetta Arcerito, Andrea Cavallaro, Marco Cavallaro, Gaetano Bertino, Isidoro Di Carlo

**Affiliations:** ^1^Department of Surgery, Taormina Hospital, 98034 Messina, Italy; ^2^Hepatology Unit, Department of Medical and Pediatric Science, University of Catania, 95100 Catania, Italy; ^3^Department of Surgical Sciences, Organ Transplantation and Advanced Technologies, University of Catania, Cannizzaro Hospital, Via Messina 829, 95100 Catania, Italy; ^4^Department of Surgery, Cannizzaro Hospital, 95126 Catania, Italy; ^5^Department of Surgery, Hamad General Hospital, P.O. Box 3050 Doha, Qatar; ^6^Endocrine Surgical Unit, “Policlinico e Vittorio Emanuele” Hospital, University of Catania, 95100 Catania, Italy; ^7^General Surgery and Senology Unit, Department of Surgery, “Policlinico e Vittorio Emanuele” Hospital, University of Catania Medical School, 95100 Catania, Italy; ^8^Radiology Unit, Guzzardi Hospital, 97019 Vittoria, Italy

## Abstract

*Introduction.* Aim of the present work is to review the literature to point out the role of laparoscopic reversal of Hartmann procedure. *Material and Methods.* Number of patients, age, sex, etiology, Hinchey classification, interval between procedure and reversal, position of the first trocars, mean operative time (min), number and causes of conversion, length of stay, mortality, complications, and quality of life were considered. *Results.* 238 males (52.4%) and 216 females (47.6%) between 38 and 67 years were analyzed. The etiology was diverticulitis in 292 patients (72.1%), carcinoma in 43 patients (10.6%), and other in 70 patients (17.3%). Only 7 articles (22.6%) reported Hinchey classification. The interval between initial procedure and reversal was between 50 and 330 days. The initial trocar was open positioned in 182 patients (43.2%) through umbilical incision, in 177 patients (41.9%) in right upper quadrant, and in 63 patients (14.9%) in colostomy site. The operative time was between 69 and 285 minutes. A total of 83 patients (12.1%) were converted and the causes were reported in 67.4%. The length of stay was between 3 and 12 days. 5 patients (0.7%) died. The complications concern 112 cases (16.4%). *Conclusion.* The laparoscopic Hartmann's reversal is safer and achieves faster positive results.

## 1. Introduction

Hartmann, a French surgeon, in 1921, first described a technique for the treatment of rectal cancer [1]. This new technique consisted in a sigmoidectomy followed by a terminal colostomy in the left iliac fossa and closure of the rectal stump. However, Hartmann never considered the possibility of restoration of the intestinal continuity [2]. In 1950, Boyden analyzed different surgical procedures for managing acute diverticulitis and presented late results with closure of the colostomy [3]. In 1993, Anderson et al. published the first report of a laparoscopically assisted Hartmann's reversal [4]. Recently, with the advent of broad-spectrum antibiotic and bowel rest, the initial treatment of diverticulitis can be a conservative approach. Laparoscopic lavage can be useful in case of small abscesses that can be drained percutaneously in case of more than 5 cm in diameter. When surgical procedure is indicated, the immediate intestinal continuity is used more and more. The only indication, as a gold standard for Hartmann procedure, remains the stercoraceous peritonitis due to a sigmoid perforation [5].

A substantial proportion of patients (up to 74%) may be left with a permanent stoma due to impossibility to restore the intestinal continuity for several and different reasons. Stoma is associated with complications and suboptimal quality of life [6]. The restoration of colonic continuity after this procedure is a challenge; reversal is associated with high morbidity rates (up to 54.8%) and significant mortality rates (up to 4%) [7]. The laparoscopic colorectal surgery to reestablish the intestinal continuity with small incisions, less postoperative pain, and earlier return to activity has been shown by many authors [4, 5, 8].

Aim of the present work is to review the literature after the first laparoscopic reversal of Hartmann procedure and to point out the role of this technique according to the actual possibilities of treatment.

## 2. Methods and Materials

**Figure 1:**
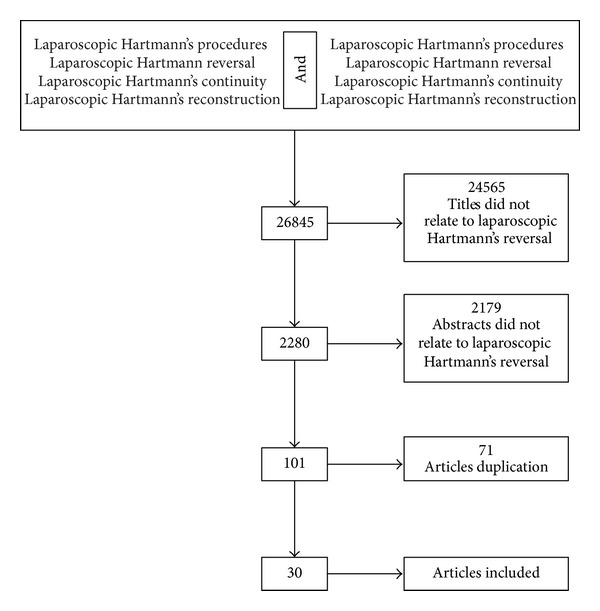
Flowchart describing the selection of studies included in this paper.

A literature search was performed using MEDLINE (PubMed), Google Scholar, and The Cochrane Library, and the articles from January 1994 until June 2013, edited in Italian, English, and French, prospective or retrospective, were analyzed. The keywords used were “laparoscopic Hartmann's procedures,” “laparoscopic Hartmann reversal,” “laparoscopic Hartmann's continuity,” and “laparoscopic Hartmann's reconstruction.” These keywords were added alone or in combination with the use of Boolean operator “AND.” Only patients with laparoscopic Hartmann reversal were considered for the review. Irrelevant articles evident from the title and abstract were excluded (Figure 1). Relevant articles referenced in these publications were obtained and the “related article” function was used to widen the results.

Outcome variables included number of patients, mean age, sex, etiology of Hartmann's procedure, Hinchey classification [9] for the first surgical procedure, interval between initial procedure and reversal (days), position of the first trocars, mean operative time (min), number of patients converted to open surgery, causes of conversion, length of stay, mortality, complications, and quality of life, which were considered for the study.

*Hinchey Classification*

 Stage I: pericolic abscess confined by the mesentery of the colon.
 Stage II: pelvic abscess resulting from a local perforation of a pericolic abscess.
 Stage III: generalized peritonitis resulting from rupture of pericolic/pelvic abscess into the peritoneal cavity.
 Stage IV: fecal peritonitis results from the free perforation of a diverticulum.

## 3. Results

The search initially yielded 26845 articles (Figure 1). After the screening of titles, 24565 articles were excluded because they were not related to laparoscopic Hartmann's reversal. After the screening of abstracts, 2179 articles were excluded because they were not about laparoscopic Hartmann's reversal. One hundred one manuscripts were screened using the inclusion criteria. A total of 71 publications were excluded because of article duplication; only 30 articles were found for the present study [4, 5, 8, 10–36]. A total of 684 patients were analyzed. The mean age was reported only in 20 articles (64.5%), and the range was between 38 and 67 years (mean 49.3 years) (Table 1). The sex was reported only in 17 articles (54.8%) for a total of 454 patients (64.7%) (Table 1). The patients were 238 male (52.4%) and 216 female (47.6%). The etiology of Hartmann's procedure was reported only in 18 articles (58.1%) for a total of 405 patients (57.7%) and was in 292 patients (72.1%) for perforated sigmoid diverticulitis, in 43 patients (10.6%) for sigmoid carcinoma, and in 70 patients (17.3%) for others causes (Table 1). Hinchey classification concerning the first intervention was sought in all the articles. The Hinchey classification was reported only in 7 articles (22.6%). In 6 articles, the Hinchey class was between III and IV, and in 1 article the Hinchey class was IV (Table 2). The range of the interval between the initial procedure and the reversal was reported only in 25 articles (83.3%) and was between 50 and 330 days (mean 163.1 days) (Table 2). In 18 articles (58.1%) the position of the first trocar for a total of 422 patients (60.1%) was reported. The initial trocar was positioned in 182 patients (43.2%) with an open port placement through an umbilical incision, in 177 patients (41.9%) in right upper quadrant, and in 63 patients (14.9%) in colostomy site (Table 2). The range of the mean operative time was reported in 28 articles (93.3%) and was between 69 and 285 minutes (mean 156.6 min) (Table 2). A total of the 83 patients (12.1%) were converted in open technique and this data was reported in all articles (100%) (Table 2). The conversion concerns only 20 articles but only in 12 of these the cause has been reported A total of 56 patients (67,4%) have been coverted to open surgery, for extensive adhesions in 39 patients (69,6%), for inadequate or lesion of the rectal stump in 4 patients (7.2%) and for other causes in 13 patients (23,2%) (Table 2). In 29 patients (34.1%) the causes of conversion were not reported. The range of the length of stay was reported in 29 articles (96.6%) and was between 3 and 12 days (mean 6.1 days) (Table 3). The mortality rate was reported in all articles (100%) and was 5 patients (0.2%) (Table 3). The complications were reported in all articles (100%) and concerned 112 cases (16.4%). They were small bowel perforation in 1 patient (0.8%), ileus in 13 patients (11.6%), rectal perforation in 1 patient (0.8%), anastomotic stenosis in 12 patients (10.7%), wound infection in 41 patients (36.6%), bleeding in 12 patients (10.7%), hematoma or abscess in 5 patients (4.7%), and other in 27 patients (24.1%) (Table 3). The quality of life of patients, after Hartmann's reversal, was not assessed in any manuscript (Table 3).

## 4. Discussion

In their most recent guidelines the American Society of Colon and Rectal Surgeons (ASCRS) stated that elective sigmoid resection after recovery from acute left-sided colonic diverticulitis should be made on a case-by-case basis [37]. This advice differs significantly from the previous advice given 6 years earlier, in which a plea for elective surgery after two episodes of diverticulitis was proposed [38]. Recent data on the natural history of diverticulitis has shown that recurrent episodes of diverticulitis mostly run a benign course, and only 5.5% of the patients with recurrent hospitalizations for diverticulitis are subjected to emergency surgery [39]. Recurrent diverticulitis even seems to reduce the risk of perforation, possibly due to adhesion formation caused by inflammation, so the number of previous episodes is no longer an indication for elective sigmoid resection [28]. Moreover, most patients who present with complicated diverticulitis experienced surgery at the time of their first attack [40].

Hartmann's procedure is usually a temporary emergency procedure for the diverticular disease. It is fast and safer operation in adverse general status and for bad local abdominal conditions. But reversal of Hartmann's procedure is associated with substantial morbidity and mortality [41].

The standard second-stage colostomy reversal to reestablish intestinal continuity requires a major abdominal operation resulting in extended recovery, incisional discomfort, and prolonged hospital stay [42]. Overall complication rates reported in series of open Hartmann's reversal range from 4% to 43%, including wound infection ranging from 5% to 24% and anastomotic dehiscence occurring in up to 12% of patients [43].

Laparoscopic advantages on open procedure to reestablish the intestinal continuity have been well demonstrated in the last twenty years; rapid postoperative recovery, less postoperative pain, earlier restoration recovery, earlier restoration of bowel function, a more rapid return to a normal diet, and reduced morbidity are the major advantages of this technique [44]. However, the advantages of laparoscopic technique do not increase the number of intestinal restorations of the continuity.

In fact, only 23% of 70% of surviving patients perform a second step with colostomy closure [45]. The reason is age-dependent; only 5% of the patients younger than 40 years remain with the stoma. But the percentage increases up to 65% in patients of 65 years and reaches 80% in patients of 80 years [46]. In this review mean age was 49.7 years. This data may be due to the fact that only younger patients decide to be submitted to Hartmann's reversal as they have a longer life expectancy and a lower rate of morbidity; there is a small prevalence of males (52.4%) in relation to the females (47.5%).

The commonest indication of Hartmann's procedure is the perforated sigmoid diverticulitis, and this is related also to this review with 70% of the patients submitted to this procedure. One of the aims of this review was to evaluate the morbidity and mortality during laparoscopic Hartmann's reversal, depending on Hinchey class during first procedure. But only seven articles reported the Hinchey class of the first surgical procedure. All these patients were Hinchey class III or IV [47].

In open surgery the patients undergoing the conventional reversal procedure less than 6 months after the initial operation have a less postoperative complications rate than those with a delay of more than 6 months. Particularly, anastomosis-related complications were 5 times more frequent [41]. Other authors believe that timing of reversal is crucial and would generally recommend a minimum wait of 6 months [23, 41]. In literature laparoscopic Hartmann's reversal after 3 months in patients that were submitted to a laparoscopic Hartmann's resection for bowel obstruction is reported [6, 14, 32]. The reversal procedure was easy with few adhesions encountered. The waiting period after the Hartmann procedure should be as short as possible and it should be decided in relation to the clinical status of the patients. Also the short period between the Hartmann and the intestinal continuity restoration is justified by the low incidence of the severe adhesions [35]. The use of sodium hyaluronate carboxymethylcellulose during the initial procedure may potentially reduce subsequent adhesion formation [2, 48], although it was not used for any patient in the reported manuscripts of the present review. Laparoscopic adhesiolysis can be particularly challenging and extensive adhesions represent the main cause of conversion in this review [8, 11, 14, 23, 27].

In the present study, there is no consensus among attending surgeons regarding the preferred approach for initial port insertion. Two techniques can be used for the first port insertion: the Veress needle (closed technique) and Hasson technique (open technique). Vascular injuries and visceral perforations are prevalently reported, respectively, with Veress and Hasson techniques [49].

Many patients after Hartmann's procedure have severe intra-abdominal adhesions. As a result, safe entry into the abdominal cavity as well as extensive laparoscopic adhesiolysis for Hartmann's reversal may be challenging. In this review, many authors, in recent years, have used the umbilical Hasson technique [28, 29, 31–33, 49]. This method allows for exploration of the abdominal cavity, feasibility assessment of the laparoscopic technique, and adhesiolysis with dissection of the colostomy under direct vision.

Due to the fact that the patients submitted to the Hartmann procedure have a previous peritonitis and the adhesions are the main cause of conversion to the open technique during laparoscopic reversal procedure, the open access through the prior colostomy seems to be the safer technique to achieve the pneumoperitoneum. This method avoids also the risk of the viscus perforation due to strong adhesions in case of umbilical Hasson technique.

An incision on the abdominal wall in the upper midline to the left of the rectus sheath near the tip of the eleventh rib in the left upper quadrant site is considered safe access too [8]. After pneumoperitoneum, two additional trocars are introduced in the upper and lower abdominal quadrants.

The mobilization of the left colon and splenic flexure are usually performed during Hartmann procedure, especially when the length of the descending colon does not permit making an easy terminal stoma. But when this procedure has not been performed during the first procedure, the laparoscopic approach has additional advantages in allowing visualization of the splenic flexure and in doing its mobilization [23].

After 8–10 weeks of the initial procedure the rectal atrophy is evident and the rectal stump is difficult to visualize [4, 18]. When the rectal stump is short, there is the possibility of injuring the bladder. To avoid this problem, some authors suggest filling the bladder with 300 mL saline solution introduced via the urinary catheter [18].

Some authors advise to leave a polypropylene suture to identify the Hartmann stump in the prior surgery or using the rectal dilators during dissection of rectal stump [8]. Others advocate using a flexible or rigid sigmoidoscope and localizing the light with the laparoscope [16]. The simple use of the stapling device inserted into the rectum to identify the rectal stump have been reported [32].

When the rectal stump is well identified a transanal end-to-end anastomosis is performed using a circular stapling device. Anastomotic integrity is confirmed easily by using insufflations of air from the rectum after overfilling the Douglas pouch with saline solution [32]; some authors request the colonscopic evaluation of the anastomosis but this is a more difficult method. The protective stoma with ileum is not performed except in very few cases related to the comorbidity of the patients [47]. In this way, the virtual ileostomy can help to minimize the complications [50]. All authors of this series have confirmed that excessive pelvic adhesions or an inability to identify the rectal stump has led to conversion rates of 12.1% [4, 5, 8, 10–36].

In open Hartmann's procedure, the mean operative time reported in literature is 167 min [51]. In laparoscopic Hartmann's procedure, the mean operative time was 171.1 min.

Many authors report less intraoperative blood loss, shorter hospital stay, less wound infection rate, less postoperative pain, lower incidence of pelvic abscess, anastomotic leak, and incisional hernia using laparoscopic reversal Hartmann's procedure [12, 27, 35]. The patient's convalescence, the first evacuation, and oral feeding are achieved faster [22]. In this review, the mean length of hospital stay was 6.2 days.

Laparoscopic reversal has shown less morbidity and mortality in relation to open Hartmann's reversal procedure. The morbidity with open Hartmann's reversal is reported at 4–43% [27], and the mortality rate ranges from 4 to 10% [22]. In the present review, morbidity was 15.8% and the mortality was 0.7%.

## 5. Conclusion

The laparoscopic Hartmann's reversal is safer and achieves faster positive results in relation to the open Hartman reversal.

## Figures and Tables

**Table 1:** Preoperative data.

Author	Year	Pz	Age (mean)	Sex		Causes		
				M	F	Perforated sigmoid diverticulitis	Perforated colon cancer	Other causes
Anderson et al. [4]	1993	2						
Gorey et al. [10]	1993	1						
Sosa et al. [11]	1994	18	38					
Costantino and Mukalian [12]	1994	3	67			3	0	0
Vernava III et al. [13]	1995	2						
Regadas et al. [14]	1996	20	52.8	10	10			
Macpherson et al. [15]	1996	12	62	5	7	9	2	1
Delgado et al. [16]	1998	11						
Köhler et al. [17]	1999	18						
Holland et al. [18]	2002	4	67			2	1	1
Vacher et al. [19]	2002	38	60			27	0	11
Mutter et al. [20]	2004	3						
Rosen et al. [8]	2005	22	54	10	12	15	2	5
Khaikin et al. [21]	2006	27	60.5	17	10	19	5	3
Golash [22]	2006	12	40	8	4	6	1	5
Slawik and Dixon [23]	2007	28	66	11	17	19	7	2
Faure et al. [24]	2007	14	61	6	8	9	4	1
Haughn et al. [25]	2008	61						
Carus et al. [5]	2008	28						
Petersen et al. [26]	2009	71		39	32			
Mazeh et al. [27]	2009	41	58.5	20	21	24	2	15
Chouillard et al. [28]	2009	44						
Agaba et al. [29]	2009	7	50	5	2	7	0	0
Svenningsen et al. [30]	2010	21	61	13	11	13	3	5
Caselli et al. [31]	2010	30	61	14	16	19	5	6
Di Carlo et al. [32]	2011	3	63	2	1	3	0	0
Huynh et al. [33]	2011	28	61	13	15	19	6	3
Leroy et al. [34]	2011	42	62.8	27	15	32	3	7
De'angelis et al. [35]	2013	28	54.9	12	16	28	0	0
Maitra et al. [36]	2013	45	59	26	19	38	2	5

Total		**684**	**987.7**	**238**	**216**	**292**	**43**	**70**
Mean		22.8	49.4	14	12.7	16.5	2.4	3.9
Range		1–71	53–67	2–27	1–32	2–38	0–7	0–15

**Table 2:** Operative data.

Author	Year	Pz	Hinchey class	Mean days after first operation	Position of first trocar			Mean operative time	Conversion	Causes		
					R Q	U T	CS			RS	EA	OT
Anderson et al. [4]	1993	2		240				—	0			
Gorey et al. [10]	1993	1						95	0			
Sosa et al. [11]	1994	18						230	4			
Costantino and Mukalian [12]	1994	3		180	3			148	0			
Vernava III et al. [13]	1995	2						195	0			
Regadas et al. [14]	1996	20		80				130	3	1	1	1
Macpherson et al. [15]	1996	12		225		10		169	0			
Delgado et al. [16]	1998	11		50				144	1			
Köhler et al. [17]	1999	18		160				114	2			
Holland et al. [18]	2002	4		83.6		4		146	1	1		
Vacher et al. [19]	2002	38		136		14	24		6			
Mutter et al. [20]	2004	3						180	0			
Rosen et al. [8]	2005	22		168				158	2			2
Khaikin et al. [21]	2006	27		255	6		21	226	4	1	3	
Golash [22]	2006	12		130	12			90	2		2	
Slawik and Dixon [23]	2007	28		330	28			80	1		1	
Faure et al. [24]	2007	14	III-IV	180		14		143	2			
Haughn et al. [25]	2008	61		240				154	8			
Carus et al. [5]	2008	28		95		28		69	5		5	
Petersen et al. [26]	2009	71		120				164	9		9	
Mazeh et al. [27]	2009	41	III-IV	187	41			193	8		3	5
Chouillard et al. [28]	2009	44		166		44		195	4			
Agaba et al. [29]	2009	7	III-IV	95		7		189	0			
Svenningsen et al. [30]	2010	21		180				285	1			
Caselli et al. [31]	2010	30	III-IV	213		30		172	3		1	2
Di Carlo et al. [32]	2011	3	IV	92		3		96	0			
Huynh et al. [33]	2011	28		135		28		166	0			
Leroy et al. [34]	2011	42	III-IV	204	42			117	4		1	3
De'angelis et al. [35]	2013	28	III-IV	134.8			18	171.1	0			
Maitra et al. [36]	2013	45			45			164.1	13	1	13	

Total		**684**		**4079.4**	**177**	**182**	**39**	**4383.2**	**83**	**4**	**39**	**13**
Mean		22.8		163.2	25.2	18.2	13	156.6	2.8	1	3.9	2.6
Range		1–71		50–330	3–45	3–44	18–24	69–285	0–13	1	1–13	1–5

Legend: RQ: right upper quadrant; UT: umbilical trocars; CS: colostomy site; RS: rectal stump; EA: extensive adhesions; OT: other.

**Table 3:** Postoperative data.

Author	Year	Pz	Length of stay	Mortality	Complication								Quality of life
					1	2	3	4	5	6	7	8	
Anderson et al. [4]	1993	2	—	0				1				1	na
Gorey et al. [10]	1993	1	5	0									na
Sosa et al. [11]	1994	18	4.3	0				1	1				na
Costantino and Mukalian [12]	1994	3	5.3	0		1							na
Vernava III et al. [13]	1995	2	4	0									na
Regadas et al. [14]	1996	20	4						2	1			na
Macpherson et al. [15]	1996	12	9	0			1	1	1			1	na
Delgado et al. [16]	1998	11	7	0									na
Köhler et al. [17]	1999	18	7.5	0					3				na
Holland et al. [18]	2002	4	7	0									na
Vacher et al. [19]	2002	38	9.5	1									na
Mutter et al. [20]	2004	3	8.5										na
Rosen et al. [8]	2005	22	4.2	0					4				na
Khaikin et al. [21]	2006	27	6	0		1			5	4		1	na
Golash [22]	2006	12	7	0									na
Slawik and Dixon [23]	2007	28	3	2					3	1		2	na
Faure et al. [24]	2007	14	9.5	0				1			1		na
Haughn et al. [25]	2008	61	4.1	0				1	2			7	na
Carus et al. [5]	2008	28	8.6	—	1			1	3				na
Petersen et al. [26]	2009	71	12	1		4				1	3	4	na
Mazeh et al. [27]	2009	41	6.5	0		3			6	1		2	na
Chouillard et al. [28]	2009	44	5	1					4			1	na
Agaba et al. [29]	2009	7	5.3	0		1		1				1	na
Svenningsen et al. [30]	2010	21	4									2	na
Caselli et al. [31]	2010	30	5.6	0				1	4				na
Di Carlo et al. [32]	2011	3	4	0									na
Huynh et al. [33]	2011	28	5	0		1				1	1	1	na
Leroy et al. [34]	2011	42	7	0				4	1	2		1	na
De'angelis et al. [35]	2013	28	6.7	0						1		3	na
Maitra et al. [36]	2013	45	6.8	0		2			2				na

Total		**684**	**181**	**5**	**1**	**13**	**1**	**12**	**41**	**12**	**5**	**27**	
Mean		22.8	6.2	0.2	1	1.9	1	1.3	2.9	1.5	1.7	2.1	
Range		1–71	3–12	1-2	1	1–4	1	1–4	1–6	1–4	1–3	1–7	

Legend: 1: small bowel perforation; 2: ileus; 3: rectal perforation; 4: problem anastomosis (stenosis, stricture, incomplete); 5: wound infection; 6: bleeding (acute, intra-abdominal); 7: hematoma/abscess; 8: other.
